# Sport-related risk factors for moderate or severe urinary incontinence in master female weightlifters: A cross-sectional study

**DOI:** 10.1371/journal.pone.0278376

**Published:** 2022-11-30

**Authors:** Marianne Huebner, Wenjuan Ma, Shirley Harding

**Affiliations:** 1 Department of Statistics and Probability, Michigan State University, East Lansing, Michigan, United States of America; 2 Department of Kinesiology, Michigan State University, East Lansing, Michigan, United States of America; 3 Center for Statistical Training and Consulting, Michigan State University, East Lansing, Michigan, United States of America; 4 Department of Osteopathic Surgical Specialties, Michigan State University, East Lansing, Michigan, United States of America; Poznan University of Physical Education, POLAND

## Abstract

**Background:**

Urinary incontinence (UI), defined as the involuntary loss of urine, is a common, multifactorial condition. It is unclear whether physical activities, their intensity or frequency, may affect the potential for UI in addition to known risk factors in the general population. Higher prevalence of UI has been observed when engaging in high-impact sports, but research is sparse regarding UI in strength sports. Since the Olympic-style weightlifting has seen an unprecedented increase in women’s participation in recent years, this study provides much-needed data to investigate whether weightlifting predisposes women to UI.

**Objectives:**

The aim was to conduct a survey of female Master athletes in countries that are members of the International Weightlifting Federation using a validated incontinence severity index and to study (1) whether known risk factors in the general population increase the odds of UI in female competitive weightlifters and (2) whether sport-related factors are associated with UI after adjusting for known risk factors. The outcomes of interest were moderate or more severe UI and incontinence during lifts specific to weightlifting competitions.

**Results:**

Respondents consisted of 824 female competitive weightlifters from 29 countries, ages 30 to 79, median 41 years. Prevalence of moderate or more severe incontinence was 32%. Higher BMI, prior pregnancies, and depressive mood increased the odds, but age was not associated. Athletes who had engaged in high-impact sports prior to starting weightlifting training were at a higher risk of UI, but participation in prior strength sports was not associated with UI. The predictive probability of moderate or more severe UI increased with more hours per week of weightlifting training.

**Conclusions:**

Our findings indicate that while female weightlifters had a higher prevalence of moderate or severe UI than in a general population according to the National Health and Nutrition Examination Survey, and that athletes who had engaged in high-impact sports prior to starting weightlifting were at a higher risk of UI.

## Introduction

Urinary incontinence (UI), defined as the involuntary loss of urine, is common. An analysis of 2015–2018 National Health and Nutrition Examination Survey (NHANES) estimated the prevalence of UI in U.S. women ages 20 and older to be 48.1% overall and 27.8% moderate or more severe [1]. The prevalence of moderate or more severe UI was lower at younger ages, e.g., 20% at ages 35–39, and higher at older ages, e.g., 40% at ages 70–74 [1]. Factors previously shown to be associated with UI are older age, higher BMI, prior hysterectomy, increasing parity, menopausal status, or higher level of anxiety or depression, lack of physical activities [1–3]. Few consistent risk factors for UI have been identified in female athletes [3], BMI, age, parity, and training hours have been considered in different sports [3, 4]. Low-level physical activities have been shown to decrease the risk of UI [1, 3, 5], but it is unclear how physical activities may influence pelvic floor muscles (PFM) and the potential for UI. PFM and an increase in UI during physical activities are believed to be influenced by an increase in abdominal pressure and exercise overload might stretch and weaken PFM [3, 6, 7]. In contrast, physical activities could strengthen PFM and co-activation of abdominal and hip muscles might alleviate UI. For example, strengthening PFM together with strengthening of hip muscles has been shown to have better results for daily urine loss than strengthening PFM alone [8]. Thus, multiple factors may balance their effect on UI in sports. Prevalence estimates of UI in female athletes differ by sport modality. Higher prevalence of UI has been observed when engaging in high-impact sports such as running (31%), ball sports (50%), or gymnastics (61%), compared to low-impact sports such as swimming (15%) or cycling but research is sparse regarding UI in strength sports [6, 9, 10].

Olympic-style weightlifting training consists of two competition lifts, the snatch and the clean & jerk, and additional exercises such as pulls and squats. The total weight lifted is influenced by lower body strength, power, speed, balance, and coordination [11]. Athletes achieve peak performance in their mid to late 20s and performance declines with increasing age with a steeper decline during the transition to menopause [12, 13]. Training for Masters athletes varies widely because of demands on time or physical and functional capacity [14]. Prior to starting with weightlifting, athletes often participated in other sports such as CrossFit, ball sports, or endurance training (e.g., running, cycling, swimming), and concurrent training is common among weightlifters [14].

Given the multifactorial condition of incontinence and the variation in training practices of Master weightlifters, we aimed to study whether known risk factors in the general population increase the odds of UI in female competitive weightlifters and, secondly, whether sport-related factors are associated with UI after adjusting for known risk factors. The main outcome of interest was moderate or more severe UI in female weightlifters.

This large transnational study contributes to the knowledge about incontinence in the sport of weightlifting. This is important, since women may not discuss UI with their coaches or are not routinely screened for UI as part of preventative care [1, 3]. The fear of visible leakage in training or on the competition platform can distress athletes and may negatively affect performance [3, 15]. This could become a more common occurrence, since the number of female weightlifters has dramatically increased in recent years and account for over 45% of the participants at World Master Weightlifting Championships [16].

## Methods

### Participants

An online survey was completed by 891 women weightlifters, ages 30 to 79, from 29 countries.

It was distributed by the Master Committee of the International Weightlifting Federation (IWF) to the National Master Chairs. They then used email or social media to communicate the study to the women members in their organization. The survey was available in four languages (English, German, French, Spanish), translated and tested by native speakers. In addition, the study was advertised in Olympic-style weightlifting interest groups via Facebook and Instagram. The survey was administered online via Qualtrics (Provo, UT, USA). All participants provided electronic informed consent prior to being able to answer survey questions. The study protocol was approved by the Michigan State University Human Research Ethics Committee (STUDY00007512).

Data collection was conducted from 25 April to 20 May 2022. Exclusion criteria were younger than 30 years (n = 1), missing age (n = 1), currently pregnant (n = 3). To account for the possibility of male participants missing responses to age of menstruation or prior pregnancies (n = 15), were also excluded. Since the focus was on competitive weightlifters, missing response to age of first competition (n = 33) or no snatch or clean & jerk total in the last 6 months (n = 14) were also exclusion criteria. This resulted in an analysis data set of 824 women [17].

### Urinary incontinence

Frequency of urine loss was characterized ‘never’, ‘less than once a month’, ‘a few times a month’, ‘a few times a week’, ‘every day or night.’ The amount of urine lost was quantified as ‘none’, ‘a few drops’, ‘small splashes’, ‘more’. The previously validated Incontinence Severity Index (ISI) was used in this investigation [18, 19] and ISI score was calculated by multiplying the frequency score by the severity score. The ISI score was categorized as ‘none’ (ISI = 0), ‘slight’ (ISI = 1–2), ‘moderate’ (ISI = 3–6), or ‘severe’ (ISI>6). Incontinence during weightlifting exercises was queried with the question *“Have you experienced urinary incontinence during training or competition*?*”*, with choices snatch, clean & jerk, pulls, or squats.

### Measures

#### Depressive mood

Symptoms of depressive mood were measured on a scale of ‘none’, ‘mild’, ‘moderate’, ‘severe’, ‘very severe’ to the question “*Which of the following symptoms apply to you at this time*? *Depressive Mood (feeling down*, *sad*, *on the verge of tears*, *lack of drive*, *mood swings)*.*”* This question is part of the validated menopausal rating scale that was part of this survey [20].

#### Menopausal status

Menopausal status was self-reported as no menstruation for a year or medically/surgically induced menopause.

#### Weightlifting performance

The best snatch and best clean & jerk performance of the last 6 months were summed. Since athletes with higher body mass typically lift more weight, it is not possible from the total weight lifted weightlifters alone to compare performance. To compare lifters in competitions the total weight lifted is adjusted for body mass and age by calculating the Sinclair-Huebner-Meltzer-Faber formula (SHMF) [21]. The Sinclair formula is a logarithmic regression model fitted to world records and Olympic Games performances to adjust for body mass [22]. This standardized total is then further adjusted to account for age associated decline [13]. While best snatch and best clean & jerk lifts may not have been achieved in the same training session or competition, the weights lifted were summed and adjusted with SHMF to represent the performance level.

#### Statistical methods

Logistic regression models were used to estimate the strength of association with moderate or more severe UI compared to none or slight of known risk factors in a general population and sport-related factors. Factors in the general population are age, BMI, prior pregnancy, menopausal status, or depressive mood. Sport-related factors examined were experience in weightlifting (start within the last 5 years, 5–10 years, or more), training hours per week, hours of physical activities per week in addition to weightlifting, performance level measured by SHMF, prior participation in high-impact sports (e.g., ball sports, gymnastics, martial arts, CrossFit), and prior participation in strength sports (e.g., powerlifting, bodybuilding, track and field throwing). Some variables were rescaled to stabilize the estimation of model parameters [23]. Age and BMI were divided by 5, thus a unit change in the model is interpreted as a change per 5 years for age and per 5 kg/m^2^ for BMI. Predictive probabilities were calculated from the model to understand the impact of training hours, performance level, prior participation in high-impact sports, and age on moderate or more severe UI while holding other variables constant (premenopausal, no depressive mood, start of weightlifting within 5–10 years, reference age 35, mean values for other variables). A p-value of 0.05 was considered statistically significant. All analyses were performed using the statistical software R version 4.0.3 [24]. The study was reported according to the STROBE statement [25].

## Results

The analysis included 824 women, ages 30 to 79. After applying exclusion criteria respondents currently lived in Africa (n = 4), Asia (n = 4), Europe (n = 117), Oceania (n = 32), or Pan-America (n = 667) which are regions recognized by the IWF.

The majority started weightlifting between the ages of 25 to 44, namely 25–29 (19.1%), 30–34 (21.7%), 35–39 (16.7%), 40–44 (12.7%). Competition experience in any sport occurred already before age 10 (30.6%) or 10–14 years (20.0%). Starting weightlifting training after age 55 occurred in 39 (4.7%) of the women. The characteristics are described in Table 1.

**Table 1:** Characteristics of respondents stratified by parity.

	N	All female weightlifters (N = 824)	Nulliparous weightlifters (N = 388)	Weightlifters with prior pregnancies (N = 436)	p
ISI	814				*χ*^*2*^_3_ = 38.76, p<0.001^1^
none		45.9%, ^374^⁄_814_	56.4%, ^217^⁄_385_	36.6%, ^157^⁄_429_	
slight		21.4%, ^174^⁄_814_	20.5%, ^79^⁄_385_	22.1%, ^95^⁄_429_	
moderate		25.3%, ^206^⁄_814_	18.7%, ^72^⁄_385_	31.2%, ^134^⁄_429_	
severe		7.4%, ^60^⁄_814_	4.4%, ^17^⁄_385_	10.0%, ^43^⁄_429_	
Age, years	824	43.8 ± 10.0	39.7 ± 8.4	47.4 ± 9.8	*F*_1 822_ = 192.9, p<0.001
Age groups	824				*χ*^*2*^_2_ = 93.95, p<0.001^1^
30–44		62.0%, ^511^⁄_824_	79.4%, ^308^⁄_388_	46.6%, ^203^⁄_436_	
45–59		29.0%, ^239^⁄_824_	16.0%, ^62^⁄_388_	40.6%, ^177^⁄_436_	
60+		9.0%, ^74^⁄_824_	4.6%, ^18^⁄_388_	12.8%, ^56^⁄_436_	
Living arrangements	823				*χ*^*2*^_3_ = 29.8, p<0.001^2^
Alone		17.9%, ^147^⁄_823_	31.0%, ^120^⁄_387_	0.6%, ^27^⁄_436_	*χ*^*2*^_3_ = 342.5, p<0.001^1^
With partner		39.6%, ^326^⁄_823_	59.4%, ^230^⁄_387_	22.0%, ^96^⁄_436_	
With family		40.8%, ^336^⁄_823_	7.5%, ^29^⁄_387_	70.4%, ^307^⁄_436_	
With others		1.7%, ^14^⁄_823_	2.1%, ^8^⁄_387_	1.4%, ^6^⁄_436_	
Height, cm	805	164.1 ± 7.5	164.5 ± 7.2	163.8 ± 7.6	*F*_1 803_ = 0.95, p = 0.331^1^
Body mass, kg	815	71.6 ± 15.6	72.5 ± 16.5	70.9 ± 14.7	*F*_1 813_ = 1.05, p = 0.306^1^
BMI, kg/m^2^	802	26.4 ± 5.1	26.5 ± 5.2	26.4 ± 5.1	*F*_1 800_ = 0.71, p = 0.399^1^
Menopause	818				*χ*^2^_2_ = 40.83, p<0.001^1^
medical		5.7%, ^47^⁄_818_	3.1%, ^12^⁄_385_	8.1%, ^35^⁄_433_	
natural		15.0%, ^123^⁄_818_	8.1%, ^31^⁄_385_	21.2%, ^92^⁄_433_	
Pre-menopausal		79.2%, ^648^⁄_818_	88.8%, ^342^⁄_385_	70.7%, ^306^⁄_433_	
Depressive Mood, moderate/severe	818	21.5%, ^176^⁄_818_	19.7%, ^76^⁄_386_	23.1%, ^100^⁄_432_	*χ*^*2*^_1_ = 1.44, p = 0.229^1^
Years active in weightlifting	824				*χ*^*2*^_2_ = 3.20, p = 0.202^1^
< 5 years		10.8%, ^89^⁄_824_	10.6%, ^41^⁄_388_	11.0%, ^48^⁄_436_	
5–10 years		45.7%, ^377^⁄_824_	48.9%, ^190^⁄_388_	42.9%, ^187^⁄_436_	
>10 years		43.4%, ^358^⁄_824_	40.5%, ^157^⁄_388_	46.1%, ^201^⁄_436_	
Hours training/week	821	7.1 ± 3.0	7.4 ± 3.2	6.8 ± 2.8	*F*_1 819_ = 6.74, p = 0.010^1^
Hours physical activity/week	819	4.3 ± 3.3	4.3 ± 3.2	4.3 ± 3.3	*F*_1 817_ = 0.69, p = 0.407^1^
SHMF (estimated)	815	185.6 ± 38.6	184.6 ± 39.3	186.4 ± 38.0	*F*_1 813_ = 0.63, p = 0.426^1^
Prior participation in high-impact sports	824	82.2%, ^677^⁄_824_	84.8%, ^329^⁄_388_	79.8%, ^348^⁄_436_	*χ*^*2*^_1_ = 3.47, p = 0.062^1^
Prior participation in strength sports	821	30.3%, ^249^⁄_821_	32.2%, ^125^⁄_388_	28.6%, ^124^⁄_433_	*χ*^*2*^_1_ = 1.24, p = 0.265^1^

ISI, incontinence severity index; BMI, body mass index; SHMF, adjusted weightlifting performance by Sinclair-Huebner-Meltzer-Faber formula

*x ± s* represents mean *±* standard deviation. *N* is the number of non-missing values. Tests used: ^1^Wilcoxon test; ^2^Pearson test.

### Prevalence of urinary incontinence

The distribution of the ISI score was ‘none’ 374 (45.9%), ‘slight’ 174 (21.4%), ‘moderate’ 206 (25.3%), ‘severe’ or ‘very severe’ 60 (7.4%). Overall, 54.1% of the athletes experienced some UI as measured by ISI>0. This was similar across age groups (52.8% in 30–44, 54.4% in 45–59, 61.1% in 60+). The proportions of athletes experiencing moderate or more severe UI were 32.6% (95% CI: 29.5, 36.0) overall and 23.1% (95% CI: 19.1, 27.7) in nulliparous women. This was similar in the Pan-American and European regions, 33.7% (95% CI: 30.1, 37.4) and 30.1% (95% CI: 22.0, 39.5), respectively.

**Fig 1:**
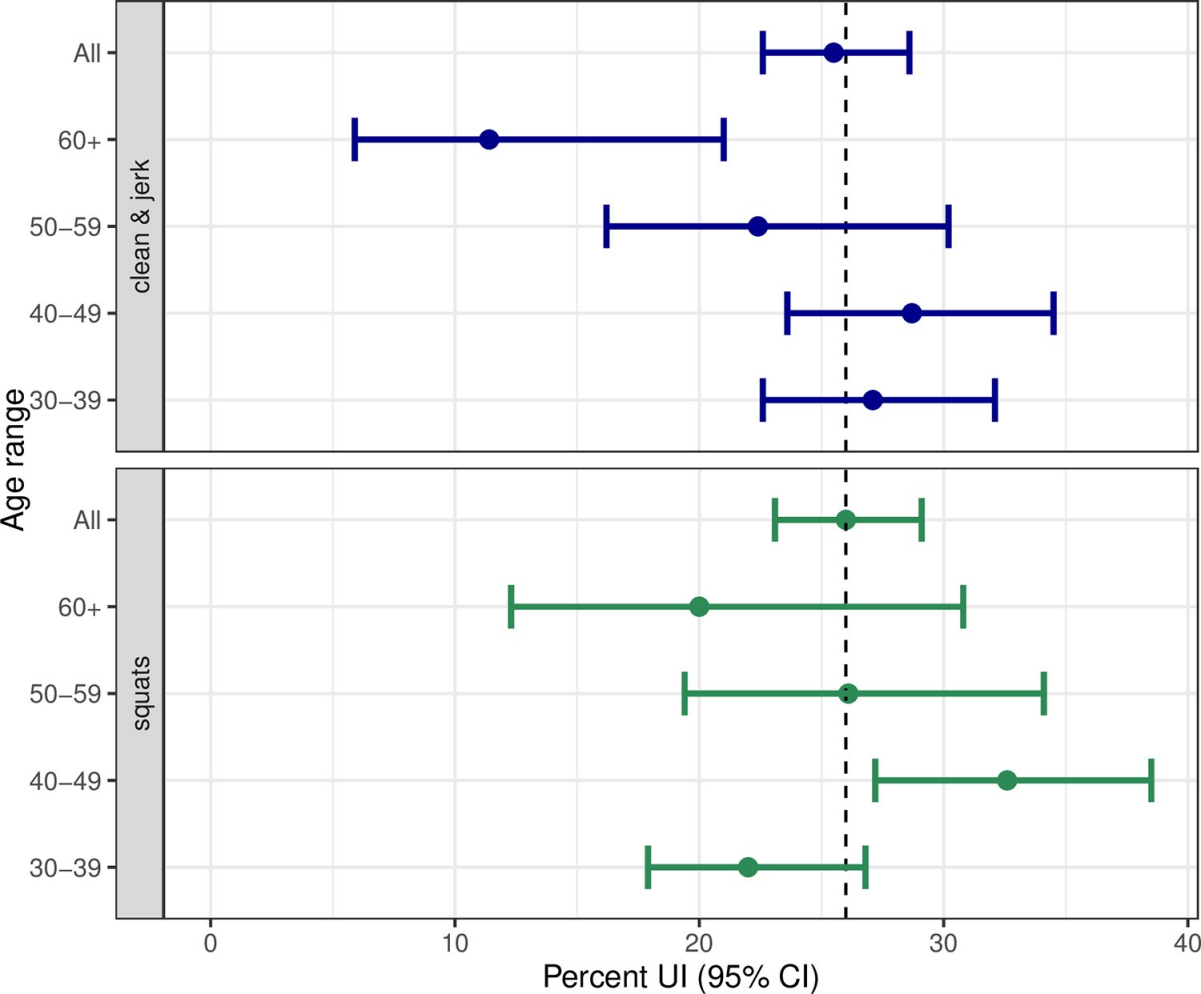
Prevalence of UI in clean & jerk and squats.

Incontinence during specific weightlifting exercises was experienced by 26.3% (n = 216) during squats, 25.6% (n = 210) during clean & jerk and occurred less during snatches 4.6% (n = 38) and pulls 5.7% (n = 47). The confidence intervals for UI during clean & jerk and squats for different age ranges are shown in Fig 1 (vertical line is at 26%).

### Risk factors for moderate or more severe urinary incontinence

**Fig 2:**
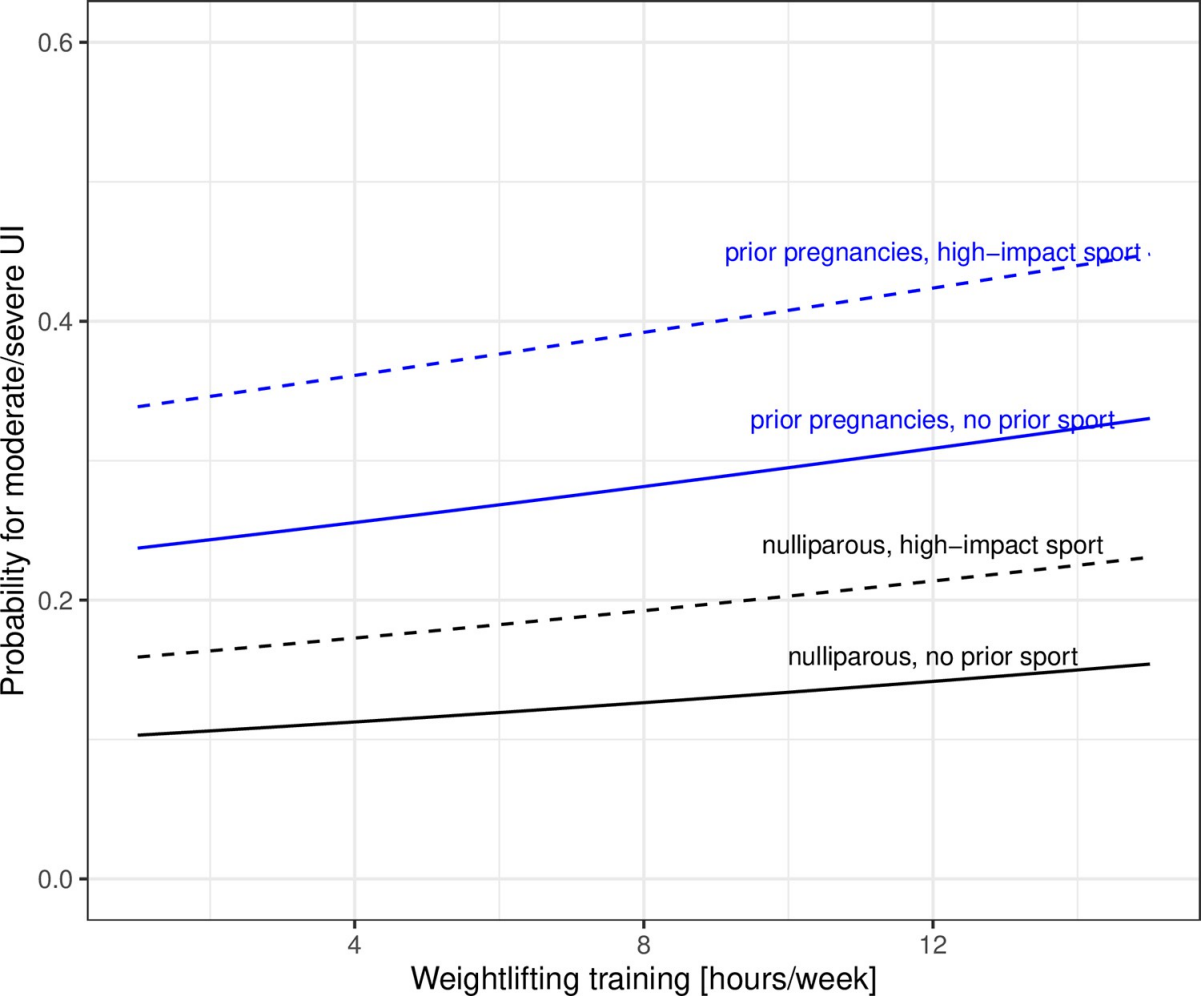
Predicted probability of UI with training hours.

**Fig 3:**
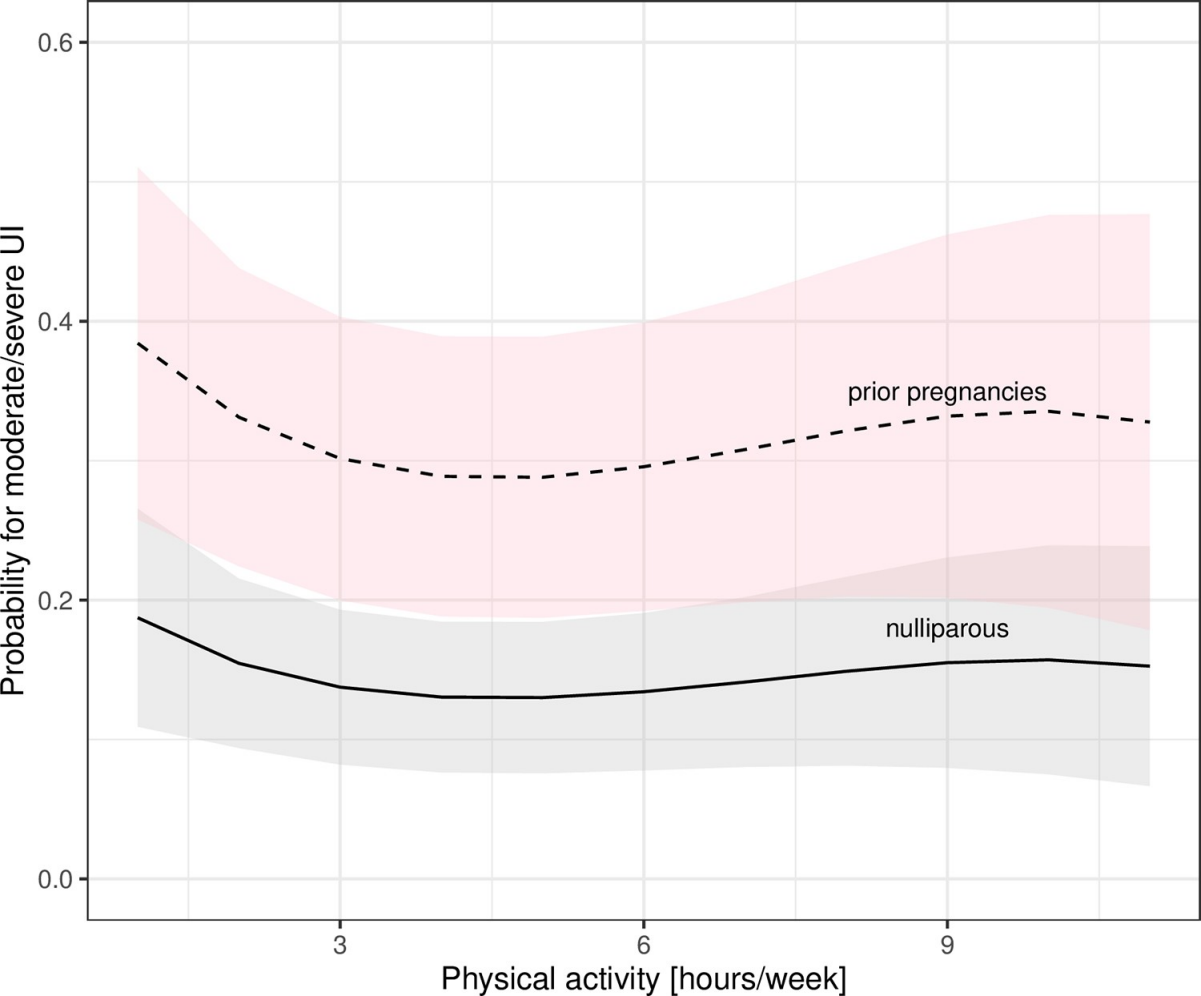
Predicted probability of UI with concurrent training.

Results from the logistic regression models are reported in Table 2. Older age was not associated with moderate or more severe UI. Higher BMI, prior pregnancies, and depressive mood were associated with moderate or more severe UI. Of the sport-related factors (hours per week weightlifting training or physical activity, prior sport participation, performance adjusted for body mass and age, experience in weightlifting) only prior participation in high-impact sports was associated with moderate or more severe UI. While hours training per week and the performance level (SHMF) were not statistically significant, there is a trend of higher probabilities with increasing hours of weightlifting training per week (Fig 2). Hours of concurrent training had a nonlinear effect, where 1 to 4 hours of additional physical activities had a lower predictive probability of UI, but an increased probability for more hours (Fig 3).

**Table 2:** Odds ratios (95% confidence intervals) for moderate or more severe UI in all female weightlifters and in the subgroup of nulliparous weightlifters.

	Moderate or more severe UI				UI during clean & jerk	
	All female weightlifters (UI = 250,N = 771)	p	Nulliparous weightlifters (UI = 83,N = 367)	p	All female weightlifters (UI = 197,N = 769)	p
	C = 0.689		C = 0.651		C = 0.687	
**General factors**						
Age [5 years]	0.96 (0.84, 1.10)	0.621	1.09 (0.89, 1.36)	0.376	0.83 (0.72, 0.96)	0.013
BMI [5 kg/m^2^]	1.25 (1.06, 1.47)	0.007	1.24 (0.97, 1.58)	0.084	1.22 (1.03, 1.45)	0.022
Prior pregnancy	2.69 (1.89, 3.84)	<0.001	---	---	2.10 (1.44, 3.07)	<0.001
Menopausal	1.07 (0.60, 1.92)	0.810	0.81 (0.27, 2.44)	0.709	1.02 (0.54, 1.92)	0.959
Depressive Mood	1.36 (1.14, 1.62)	<0.001	1.49 (1.13, 1.99)	0.005	1.32 (1.10, 1.59)	0.003
**Sport-related factors**						
Years active in weightlifting						
< 5 years	reference		reference		reference	
5–10 years	1.05 (0.61, 1.83)	0.837	1.32 (0.53, 3.30)	0.552	0.96 (0.52, 1.75)	0.884
> 10 years	1.41 (0.81, 2.46)	0.220	1.06 (0.41, 2.77)	0.899	1.56 (0.87, 2.92)	0.128
Weightlifting training [hours/week]	1.03 (0.98, 1.09)	0.238	1.05 (0.96, 1.14)	0.295	1.06 (0.95, 1.22)	0.073
Physical activity^1^	0.67 (0.47, 0.95)	0.024	0.74 (0.43, 1.28)	0.282	0.68 (0.46, 1.01)	0.056
PA^2	1.07 (1.00, 1.13)	0.034	1.03 (0.94, 1.28)	0.517	0.68 (0.46, 1.01)	0.017
PA^3	1.00 (0.99, 1.00)	0.039	1.00 (0.99, 1.00)	0.679	1.09 (1.02, 1.17)	0.009
SHMF	1.01 (0.96, 1.05)	0.753	1.05 (0.98, 1.13)	0.156	0.99 (0.99, 1.00)	0.002
Prior participation in high-impact sports	1.63 (1.03, 2.58)	0.036	1.24 (0.57, 2.65)	0.588	1.08 (0.66, 1.75)	0.757
Prior participation in strength sports	0.77 (0.54, 1.00)	0.148	0.93 (0.53, 1.62)	0.795	0.79 (0.54, 1.15)	0.218

BMI, body mass index; SHMF, adjusted weightlifting performance by Sinclair-Huebner-Meltzer-Faber formula

^1^PA = physical activity [hours/week] (linear, quadratic, cubic)

Nulliparous women with depressive mood were twice as likely to experience moderate/severe UI than nulliparous women with no or mild depressive mood. While BMI was not statistically significant in nulliparous women, the confidence intervals are almost the same as in the overall cohort. No other factors were associated with moderate or more severe incontinence.

### Risk factors for urinary incontinence during clean & jerks

Risk factors for UI during clean & jerk were higher BMI, prior pregnancies, and depressive mood (Table 2). Prior participation in high-impact or strength sports was not associated. For older athletes there was a decreased risk of UI in clean & jerk but not in squats (Table 2 and Fig 1). Odds ratios for higher performance levels and longer training hours were 1.08 (95% CI: 1.03, 1.14) and 1.06 (95% CI: 0.95, 1.22), respectively. Physical activity levels had a non-linear effect with few hours decreasing the probability of UI during clean & jerk and more hours increasing the probability (Fig 3).

## Discussion

This large transnational study of 824 Master female weightlifters from 29 countries contributes to the knowledge about UI in the sport of weightlifting. This is important, since women may not discuss UI with their coaches or are not routinely screened for UI as part of preventative care [1, 3]. Multiple factors play a role in UI in women. While low level physical activity has been shown to alleviate the risk of UI, the prevalence in female athletes, especially in high-impact sports, is higher than the prevalence for women in general. Weightlifting training requires lower body strength, power, speed, balance, and coordination [11]. Master weightlifters also engage in concurrent training to varying degrees. Little is known about sport-related risk factors for UI after adjusting for known risk factors in general populations.

The widely used incontinence severity index [18] was used to determine the moderate or more severe incontinence by considering the combination of frequency and amount of leakage.

The main findings of this study were that (1) the prevalence of moderate or more severe incontinence in weightlifters (32%) was higher on average than in a general population of the recent NHANES study (20% to 27% at different ages); (2) higher BMI, prior pregnancies, and depressive mood increased the odds, but age and menopausal status was not associated; (3) athletes who had engaged in high-impact sports prior to starting weightlifting training were at a higher risk of UI, but participation in prior strength sports was not associated with UI; (4) the predictive probability of moderate or more severe UI increased with more hours per week of weightlifting training, but physical activities in addition to weightlifting could reduce the probability.

### Prevalence of urinary incontinence in female weightlifters

In this cohort of female weightlifters, ages from 30 to 79 years with average age 44 ± 10 years, the prevalence of moderate or more severe UI was higher in the weightlifters than in the NHANES population study, 32.6% in weightlifters compared to 19.5% for ages 30–39 increasing to 26.5% for ages 60 to 69 in NHANES. Leakage during weightlifting exercises was highest during squats and clean & jerk (26%) compared to snatch or pulls (4–6%). This corroborates the findings of the weightlifting study by Wikander and colleagues [15]. The prevalence of any UI (i.e., including slight, moderate, more severe) was 54.1% overall. This is comparable or lower than the prevalence in a recent NHANES of adult US women estimated to be 51.9% for ages 30–39 and increasing to 71.9% in ages 60 and older [1]. In contrast, two weightlifting studies with 191 and 53 female weightlifters, respectively, found a lower prevalence of any UI, 31.9% [15] and 36% [26]. There is a large volume of literature that estimates a higher prevalence due to sport participation, especially in high-impact sports, but the wide range of estimates between studies may result from differences in instruments to measure UI or differences in demographics of participants [3, and references therein]. Most Master weightlifters in this cohort participated in high-impact sports prior to starting weightlifting, 80% in those under age 60 and 63% in those 60 years or older. Prior participation in CrossFit, ball sports, endurance (e.g., running, cycling, swimming) are common among competitive Master weightlifters [14]. Women older than 60 years started weightlifting later in life, between ages 55 and 59 years. Thus, sport history should be considered a factor in the prevalence of UI in Master weightlifters.

### Risk factors for urinary incontinence in female weightlifters

Factors previously shown to be associated with UI in general populations are older age, higher BMI, prior hysterectomy, increasing parity, menopausal status, higher levels of anxiety or depression, or lack of physical activity [1]. It is important to consider whether known risk factors of UI in the general population also apply in athletes. For example, age was not associated with UI in former Olympians [3, 27].

### Do known risk factors in general populations increase urinary incontinence in female weightlifters?

In female weightlifters higher BMI, prior pregnancy, and depressive mood were associated with moderate or more severe UI or with UI during clean & jerk in training or competition. This is aligned with findings of the general population in the US [1]. In previous studies on weightlifters and powerlifters both BMI and prior pregnancy were correlated with UI [15, 28].

We did not find an association of age with moderate or severe UI in weightlifters. This finding could support the postulate that physical activity can be preventative of UI at older ages [3, 8]. This agrees with the conclusion that lifting heavy weights will not exacerbate UI over time in a prior study [15]. Older women in general populations may lead more sedentary lifestyles or are more likely to be obese and thus would be at risk for UI at older ages [1]. Another interpretation could be that older women with UI discontinue participation in the sport of weightlifting or are deterred from starting weightlifting. However, since older age was associated with a decreased risk for UI during clean & jerk but not for squats, this could indicate that the training program at older ages is different than those of younger athletes with fewer repetitions and lighter loads relative to their body mass and age for the competition lifts, such as clean & jerk, but continuing with higher repetitions and loads for the squats [15]. A future study with training diaries would be helpful to describe training intensity and volume at different ages.

In this study severe or very severe symptoms of depressive moods were identified by 5.0% of the respondents and moderate symptoms by 16.6%. These were significantly associated with UI. This has not been considered in previous studies on UI in athletes. In general, the prevalence of depression in U.S. women older than 20 years was 10.4% during 2013–2016 according to the Center for Disease Control (CDC). Depression also occurs in athletes [29]. Performance goals, insufficient recovery times, or injuries could increase the incidence of depression in athletes. Since weightlifting is a sport with body weight categories, weight loss or weight gain before competitions could also contribute. Previous research indicated that depression affected weightlifting training in 5.2% of the women [30].

### What sport-related factors increase urinary incontinence?

Performance level as measured by SHMF was not associated with moderate or severe UI, but higher performance levels were associated with UI during clean & jerk. This corresponds to studies by Wikander and colleagues [15] who noted that competition total was not correlated with ISI scores, but that heavier loads were risk factors for UI in weightlifting exercises [15, 28]. Of the two competition lifts, the weight lifted in clean & jerk were on average 28% higher than the weight lifted in snatches. Athletes with higher performance levels lift heavier loads in the clean & jerk relative to their body mass and age.

Prior participation in high-impact sports was associated with UI, while prior participation in strength sports was not associated with UI. Whether prior sport participation predisposes women to UI later in life is not well studied in the literature. Female athletes in high-impact sports have a higher prevalence of UI [6], but one study showed that there were no differences in UI prevalence between a high-impact group (former gymnasts and track and field) and a low-impact group (swimmers) 20 years after they competed in the Olympics [27]. Our study cohort consists of currently active weightlifters who may have a shorter length of time since discontinuation of high-impact sport activities, or that a proportion of weightlifters include such activities in concurrent training as noted in a previous study on training habits of weightlifters [14].

An increase in weightlifting training hours resulted in a larger predictive probability of UI. Stress incontinence is a type of UI that is associated with physical exertion. It is associated with activities such as straining or exercise which place stress on periurethral supportive tissue. Thus, longer hours of weightlifting training could be associated with more repetitions of lifting heavier weights and thus cause UI to become worse. In contrast, predictive probability of UI decreased when engaging in a few hours of additional physical activities, but extended hours of physical activities could adversely affect the probability of UI. While we did not distinguish which physical activities were pursued in addition to weightlifting training; it is likely that a few hours refer to active recovery between training units, such as walking, while longer hours may be due to engagement in other sports such as CrossFit. There are conflicting results in the literature about training hours per week, showing no association in nulliparous athletes [3, 31] compared to an increase in UI in a small study of 50 female athletes [32] or a correlation between urge incontinence and more than 7 hours of training per week in different sports [4]. This cohort of Master weightlifters trained an average of 7 hours per week in weightlifting and engaged in additional physical activities. Discrepancies in studies may be due to the age range, type of sport, such as in powerlifters with higher repetitions and heavier loads [28] or due to variability in training programs and sport history. To the best of our knowledge concurrent training has not been considered in other studies on UI although it is a common practice in weightlifting and other sports [14].

A limitation is that this is an observational study with self-reported data, and thus causality could not be established. Longitudinal studies would be helpful to estimate the incidence of UI in athletes over time. Information on medication use such as diuretics that could worsen incontinence was not available. However, diuretics are included on World Anti-Doping Agency’s (WADA) list of prohibited substances among other classes of drugs [33]. We did not distinguish the mechanism of UI, such as stress UI, defined as “involuntary loss of urine on effort or physical exertion, or with sneezing or coughing” or urge UI, defined as “complaint of involuntary loss of urine associated with urgency”. However, we examined leakage during weightlifting exercises which is an aspect of stress UI. Strengths of this study are the large participation of Master athletes from 29 IWF countries and using a validated incontinence severity index which encompasses both frequency and amount of leakage that has been widely used in many populations including strength athletes [2, 15]. Since the sport of weightlifting has seen an unprecedented increase in women’s participation, this study provides much-needed data to investigate whether Olympic-style weightlifting predisposes women to UI. Research on weightlifting and UI is limited. New in our study is the systematic evaluation of sport-related factors such as hours per week of weightlifting training and of concurrent training and evaluating the association of depressive mood with UI in athletes.

## Conclusions and implications

Our findings indicate that while female weightlifters had a higher prevalence of moderate or severe UI than in a general population (NHANES), that athletes who had engaged in high-impact sports prior to starting weightlifting were at a higher risk of UI, and that depressive mood was a risk factor for UI in athletes. Sport-related factors, such as higher performance levels and longer hours of exercise, were associated with UI during clean & jerk.

An assessment of UI would be valuable for athletes who had participated in high-impact sport. Education and information for coaches and athletes to increase knowledge about UI may open the door for communication about this condition to optimize training and improve UI [3, 34]. Specifically, deliberate practice of PFM (Kegel exercises) and exercises with hip muscle co-activation and choices in additional physical activities may alleviate or even reverse symptoms. Dietary choices such as fluid intake, in particular caffeine or alcohol, could be a factor in UI [15]. It is important to assess and manage fluid intake based on time to both prevent dehydration and manage UI. Other options include placement of pessary or tampons during training or sling surgery approaches [7]. Finally, athletes are not immune to depression. Clinicians must be aware of the impact of antidepressant medication on UI or on lower urinary tract function [2, 35]. Expectations for maximum performances, injuries, or targeting a specific body mass could worsen depressive moods. Thus, awareness of mental health care options for athletes is important.
